# Dystrophin is expressed in smooth muscle and afferent nerve fibers in the rat urinary bladder

**DOI:** 10.1002/mus.26518

**Published:** 2019-06-07

**Authors:** Judith M. Lionarons, Govert Hoogland, Ruben G. F. Hendriksen, Catharina G. Faber, Danique M. J. Hellebrekers, Gommert A. Van Koeveringe, Sandra Schipper, Johan S. H. Vles

**Affiliations:** ^1^ Department of Neurology Maastricht University Medical Center PO Box 5800, 6202 AZ Maastricht The Netherlands; ^2^ School for Mental Health & Neuroscience Maastricht University Maastricht The Netherlands; ^3^ Department of Neurosurgery Maastricht University Medical Center Maastricht The Netherlands; ^4^ Department of Urology Maastricht University Medical Center Maastricht The Netherlands

**Keywords:** Duchenne muscular dystrophy, dystrophin, human urinary bladder, lower urinary tract symptoms, maturation, micturition, rat urinary bladder

## Abstract

**Introduction:**

With increasing life expectancy, comorbidities become overt in Duchenne muscular dystrophy (DMD). Although micturition problems are common, bladder function is poorly understood in DMD. We studied dystrophin expression and multiple isoform involvement in the bladder during maturation to gain insights into their roles in micturition.

**Methods:**

Dystrophin distribution was evaluated in rat bladders by immunohistochemical colocalization with smooth muscle, interstitial, urothelial, and neuronal markers. Protein levels of Dp140, Dp71, and smooth muscle were quantitated by Western blotting of neonatal to adult rat bladders.

**Results:**

Dystrophin colocalized with smooth muscle cells and afferent nerve fibers. Dp71 was expressed two‐ to threefold higher compared with Dp140, independently of age. Age‐related muscle mass changes did not influence isoform expression levels.

**Discussion:**

Dystrophin is expressed in smooth muscle cells and afferent nerve fibers in the urinary bladder, which underscores that micturition problems in DMD may have not solely a myogenic but also a neurogenic origin. *Muscle Nerve* **60**: 202–210, 2019

**Abbreviations**
aSMA: α‐smooth muscle actin
AQP: aquaporin
CGRP: calcitonin gene‐related peptide
DAB: 3,3′‐diaminobenzidine
DGC: dystrophin‐glycoprotein complex
DMD: Duchenne muscular dystrophy
GAPDH: anti‐glyceraldehyde‐3‐phosphate dehydrogenase
K_IR_: inward rectifying potassium channel
LP: lamina propria
LUTS: lower urinary tract symptoms
ML: smooth muscular layer
PBS: phosphate‐buffered saline
PGP 9.5: protein gene product 9.5
RT: room temperature
VIM: vimentin

Duchenne muscular dystrophy (DMD) is a dystrophinopathy caused by X‐linked recessive mutations in the *DMD* gene, generally resulting in absence of the dystrophin protein.^1^ Although DMD is characterized by progressive muscle weakness, it is not solely a muscle disorder. Physical (i.e., genitourinary and gastrointestinal) as well as mental (i.e., cognitive and behavioral) comorbidities have been reported.^2^ Because of improved care, the life expectancy of patients has increased significantly over the past few decades.^3^ As a result, comorbidities such as lower urinary tract symptoms (LUTS) are now becoming clinically significant.^4^, ^5^ In an outpatient rehabilitation population, 85% of patients with DMD reported 1 or more LUTS ranging from decreased bladder sensation, nocturia, and incontinence to retention and decreased flow.^4^ Others have found similar prevalence rates of at least 1 LUTS (71.4%) in muscular dystrophies.^5^ Despite a high prevalence of LUTS, especially as patients grow older, genitourinary functioning has not been well investigated.

Dystrophin is an anchoring protein that links the intracellular cytoskeleton to transmembrane components of the dystrophin‐glycoprotein complex (DGC), which is responsible for the stabilization of the muscle cell during contraction.^6^, ^7^ Dystrophin is not expressed exclusively in striated muscle but also in other tissues (i.e., brain, retina, kidney).^8^ Recently, dystrophin was found in smooth muscle cells of normal human bladder tissue.^9^

The expression of dystrophin in multiple organs possibly determines the occurrence and severity of comorbidities in DMD. The *DMD* gene produces a range of different transcripts encoding various dystrophin isoforms ranging in size with molecular weights of 71, 116, 140, 260, and 427 kDa. The main isoform found in striated muscle tissue is known as the *full‐length isoform*, Dp427m.^7^ Shorter isoforms Dp71 and Dp140 are particularly of interest in relation to micturition pathophysiology because of their expression in smooth muscle tissue as well as in the nervous system.^8^, ^10^ Although the effects of *DMD* gene mutations on striated muscle have been well established, their effects on smooth muscle and expression in the bladder are unclear. During development, structure and distribution of bladder smooth muscle and urothelial cells undergo changes to permit functional adaptation.^11^ Morphological changes could be accompanied by alterations in dystrophin expression.^12^ In addition, changes occurring in the cellular localization of other DGC proteins in DMD are a secondary consequence of the absence of dystrophin.^7^ Having considered these morphological changes and the occurrence of age‐related LUTS in DMD, we sought to evaluate dystrophin isoform expression during postnatal development of the healthy rat urinary bladder.

## MATERIALS AND METHODS

### Ethical Statement

All procedures were performed in compliance with Maastricht University's Medical and Animal Ethical Committee guidelines. All materials used in this study were obtained from *post mortem* animals that had been humanely killed for other experiments. Consequently, no ethical approval was required. Human bladder tissue samples from patients with an enlarged prostate, which were obtained intraoperatively on the basis of clinical indication and used for other research purposes, were provided by the Department of Urology of Maastricht University Medical Center (approval protocol NL45847.068.13).

### Immunohistochemistry

Immediately after decapitation, bladders and biceps femoris muscle were removed from naive 6‐month‐old male Sprague‐Dawley rats and immersion fixed in 0.1 M phosphate buffered saline (PBS) containing 4% paraformaldehyde for 1 h at room temperature (RT). Tissue cryoprotection was established with 10% and 20% sucrose/0.1 M PBS for 24 h. Next, tissue samples were rapidly frozen by using CO_2_ and stored at −80°C. After intraoperative resection, 1 human bladder tissue sample of a 59‐year‐old male patient was prepared in a similar manner.

For immunohistochemistry, 10‐μm transversal sections were cut on a cryostat. Standard hematoxylin‐eosin (Merck, Darmstadt, Germany) staining was performed to evaluate the general histology of the specimen. Then, 3,3′‐diaminobenzidine (DAB; Sigma‐Aldrich, St Louis, Missouri) staining was performed to evaluate regional dystrophin distribution in human bladder tissue samples. Antigen retrieval was performed by placing sections in sodium citrate buffer (10 mM sodium citrate, 0.05% Tween 20) in a microwave oven for 15 min at 95°C–100°C. Endogenous peroxidase activity was blocked with 0.3% hydrogen peroxide (H_2_O_2_) for 30 min at RT. Nonspecific antibody binding was blocked with 10% normal donkey serum (Millipore, Etobicoke, Ontario, Canada) in PBS containing 0.01% Triton X‐100 for 1 h at RT. Sections were then incubated with 1:200‐diluted polyclonal rabbit anti‐dystrophin primary antibody (Ab15277; Abcam, Cambridge, United Kingdom) for 40 h at 4°C, followed by 1:200‐diluted donkey anti‐rabbit biotinylated secondary antibody (Jackson Immunoresearch, Cambridgeshire, United Kingdom) for 2 h at RT. Next, 1:400‐diluted avidin‐biotin complex (ABC; Vector Laboratories, Peterborough, United Kingdom) was used for 1.5 h at RT. Finally, sections were incubated with DAB substrate buffer containing 1% DAB, 0.05 M Tris hydrochloride, 1% nickel chloride and 0.3% H_2_O_2_ for 10 min at RT. After this, regional dystrophin distribution was evaluated by using 1:200‐diluted polyclonal rabbit anti‐dystrophin in rat bladder tissue samples, as described previously.^13^ For colocalization immunohistochemistry, 1:200‐diluted polyclonal rabbit anti‐dystrophin was used together with (1) undiluted monoclonal mouse anti‐α‐smooth muscle actin (aSMA; ready to use; Dako, Glostrup, Denmark) as a marker of smooth muscle cells; (2) 1:1,000‐diluted monoclonal mouse anti‐vimentin (VIM; MU074‐UC; BioGenex, Fremont, California) as a marker of interstitial cells; (3) 1:3,000‐diluted polyclonal chicken anti‐protein gene product 9.5 (PGP 9.5; Ab10404; Abcam) as an urothelial marker; (4) 1:200‐diluted polyclonal goat anti‐calcitonin gene‐related peptide (CGRP; Ab36001; Abcam) as a neuronal marker. In addition, 1:100‐diluted polyclonal rabbit anti‐aquaporin 3 (AQP‐3; AQP‐003; Alomone, Jerusalem, Israel) was used as an AQP marker on its own because it was produced in the same host as the anti‐dystrophin antibody causing cross‐reactivity. Sections were incubated simultaneously with anti‐dystrophin primary antibody for 40 h and 16 h for all other primary antibodies at 4°C, followed by 1:100‐diluted donkey anti‐rabbit Alexa Fluor 488 or 594 secondary antibodies (Invitrogen, Carlsbad, California) for 2 h at RT. For the markers, sections were incubated with the following secondary antibodies for 2 h at RT: (1) 1:100‐diluted donkey anti‐rabbit Alexa Fluor 594 secondary antibody for aSMA and VIM; (2) 1:100‐diluted goat anti‐chicken Alexa Fluor 488 secondary antibody (Thermo Fisher Scientific, Eugene, Oregon) for PGP 9.5 and CGRP; and (3) 1:200‐diluted goat anti‐rabbit Alexa Fluor 488 secondary antibody (Thermo Fisher Scientific) for AQP‐3. After this, sections were additionally stained with Hoechst (diluted 1:500; 15 min at RT; Sigma‐Aldrich) to visualize cell nuclei.

Photo micrographic images were made by using a disk‐spinning unit‐microscope connected to an Olympus XC10 camera (Olympus, Tokyo, Japan). Images were taken with fluorophore‐dependent exposure times that were determined by detection limits in the negative controls (without primary antibody). Exposure times were filter‐specific (Hoechst 50 ms, Texas Red® 700 ms, and Fluorescein isothiocyanate 500 ms). In addition, confocal 0.5‐μm space Z‐stacks were made of urothelial, interstitial, and smooth muscle cells.

### Western Blotting

Naive male 24‐h‐, 2‐week‐, 4‐week‐, and 6‐month‐old Sprague‐Dawley rats were humanely killed by decapitation. Bladder and biceps femoris (positive control) muscle were immediately dissected and snap frozen in liquid nitrogen and then stored at −80°C.

Because the quantitation of dystrophin by Western blotting has been shown to be highly accurate, each bladder was briefly homogenized in lysis buffer (1 g tissue per 9 ml lysis buffer), containing 0.01 M PBS, 1% IGEPAL (Sigma Aldrich), 0.1% Triton X‐100, 1 mM ethylene glycol tetra acetic acid, and 1 mM ethylenediaminetetraacetic acid.^14^ Next, protein concentrations of the homogenates were measured with a Bradford protein assay using a bovine serum albumin calibration curve (BioRad, Hercules, California^15^). Proteins (20 and 10 μg per sample for dystrophin and aSMA, respectively) were resolved by 10% (dystrophin) and 7.5% (aSMA) polyacrylamide gel electrophoresis. Each sample was run (4 h, 90 V, 4°C) in duplicate and then transferred (16 h, 90 mA, 4°C) to a Polyvinylidene fluoride membrane (Merck Millipore, Billerica, Massachusetts). After transfer, membranes were subsequently incubated in Odyssey blocking buffer (LI‐COR, Homburg, Germany) for 2 h at RT in polyclonal rabbit anti‐dystrophin primary antibody (diluted 1:200; Ab15277; Abcam) or monoclonal mouse anti‐aSMA primary antibody (diluted 1:50; ready‐to‐use, mouse IgG concentration 70 mg/L (total protein concentration 4.2 g/L); Dako) overnight at 4°C and finally in goat anti‐rabbit IRDye 800CW for dystrophin (diluted 1:10,000; LI‐COR) or donkey anti‐mouse IRDye 680RD for aSMA (diluted 1:10,000, LI‐COR) for 1 h at RT. The anti‐dystrophin antibody that was used binds to amino acid 3650 at the C‐terminal. The C‐terminal is preserved in all isoforms, and, therefore, the antibody is used for detection of different isoforms with varying protein sizes, as previously described.^13^ An overview of the different dystrophin isoforms along with the epitope targeted by the antibody is presented in Supporting Information Data. As a loading control, membranes were simultaneously stained with anti‐glyceraldehyde‐3‐phosphate dehydrogenase (GAPDH) primary antibody (diluted 1:2,000,000; Fitzgerald Industries International, Acton, Massachusetts) and donkey anti‐mouse IRDye 680RD secondary antibody (diluted 1:10,000; LI‐COR). All antibodies were diluted in Odyssey blocking buffer. For aSMA, the membrane was cut above 37 kDa before antibody incubations, separating aSMA (42 kDa) and GAPDH (37 kDa) because both antibodies were produced in the same host causing cross‐reactivity. Immunoreactive protein bands were visualized by an Odyssey infrared imaging system (LI‐COR Biosciences, Lincoln, Nebraska^16^) and quantitated by a blinded observer in ImageJ.^14^ Optical density pixel intensities were measured as described previously.^13^, ^17^ Expression levels were normalized to the optical density of the respective GAPDH immunoreactive band.

### Data Analysis

The expression level of each sample was calculated as the average of 1 duplicate. These values were then used to calculate a mean ± SEM per age group. To assess potential effects of maturation on dystrophin isoform expression, we compared the 4 different age groups by analysis of variance, followed by a least significant difference *post hoc* test in SPSS Statistics (IBM, Armonk, New York). Differences between groups were considered statistically significant at *P* < 0.05.

## RESULTS

### Dystrophin Distribution in the Urinary Bladder

**Figure 1:**
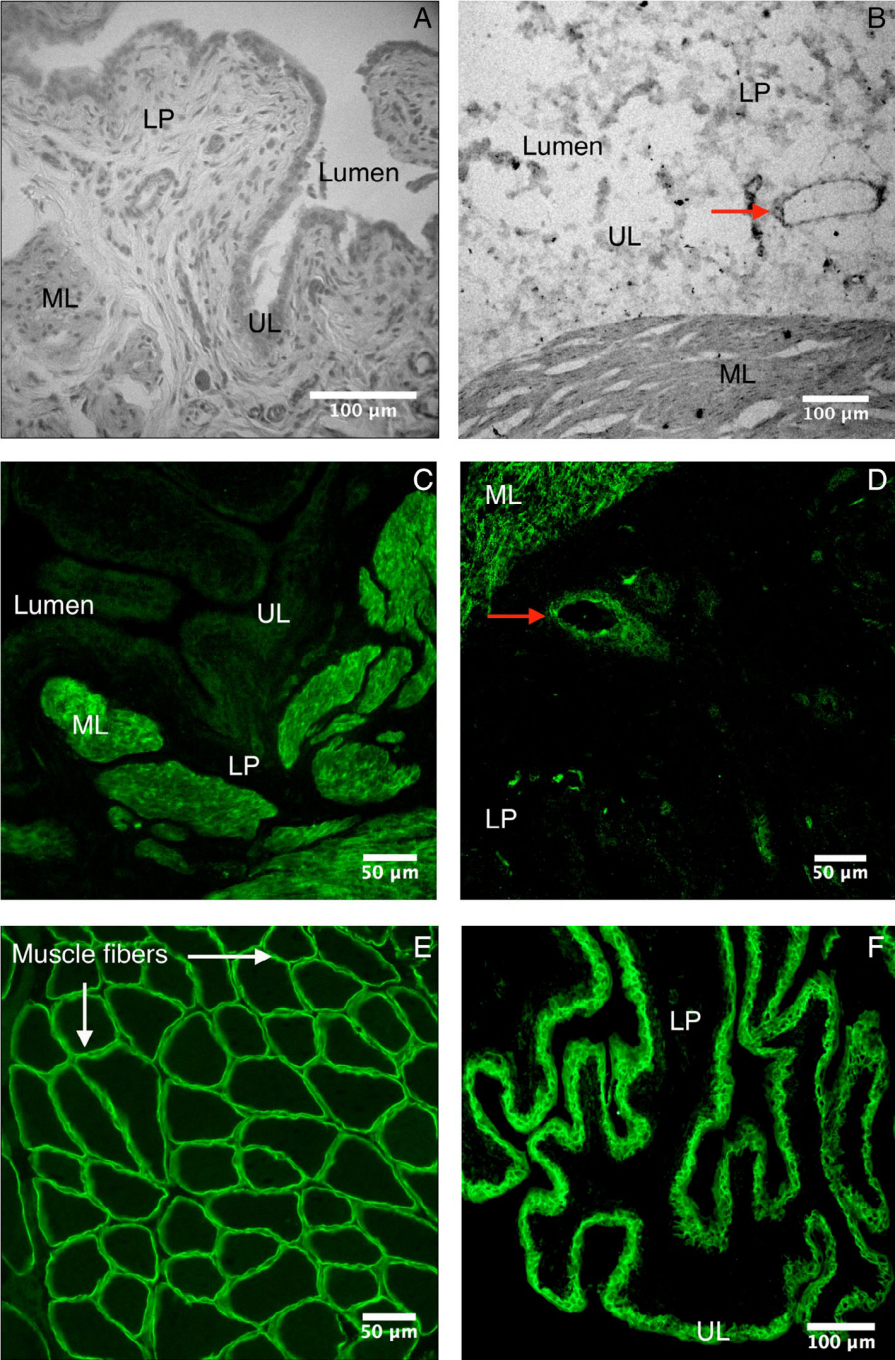
Histological and immunohistochemical staining of the human and rat bladder and rat biceps femoris muscle. **(A)** Hematoxylin and eosin staining of the rat bladder, 10‐μm transverse sections, ×100. **(B)** Human bladder immunohistochemically stained for dystrophin, 10‐μm transverse sections, ×200. **(C,D)** Rat bladder immunohistochemically stained for dystrophin, 10‐μm transverse sections, ×100, ×200, respectively. Dystrophin is predominantly expressed in the smooth muscular layer (**B,C**). The staining pattern furthermore suggests dystrophin expression in blood vessels in the LP (red arrow, **B,D**). **(E)** Rat biceps femoris muscle fibers immunohistochemically stained for dystrophin as a positive control, 10‐μm transverse sections, ×200. **(F)** Rat bladder immunohistochemically stained for aquaporin 3, 10‐μm transverse sections, ×100. Scale bars = 100 μm in A,B,F; 50 μm in C‐E. LP, lamina propria; ML, smooth muscular layer; UL, urothelial layer. [Color figure can be viewed at wileyonlinelibrary.com]

We identified the following bladder structures with hematoxylin‐eosin staining: a urothelial layer, lamina propria (LP), and smooth muscular layer (ML; Fig. 1A). Interstitial cells are localized in the LP and within the ML.^18^ Analysis of regional dystrophin distribution showed its ubiquitous expression in the longitudinal and circular ML of human as well as rat bladder tissue (Fig. 1B,C). The observed staining pattern furthermore suggested dystrophin expression in blood vessels (Fig. 1B,D). As a positive control, rat skeletal muscle showed a strong immunoreactive signal for dystrophin at the sarcolemma (Fig. 1E). Negative controls (omitting secondary antibodies) showed no detectable signal when sections were evaluated with the same exposure times as those used for dystrophin stained sections (not shown).

**Figure 2:**
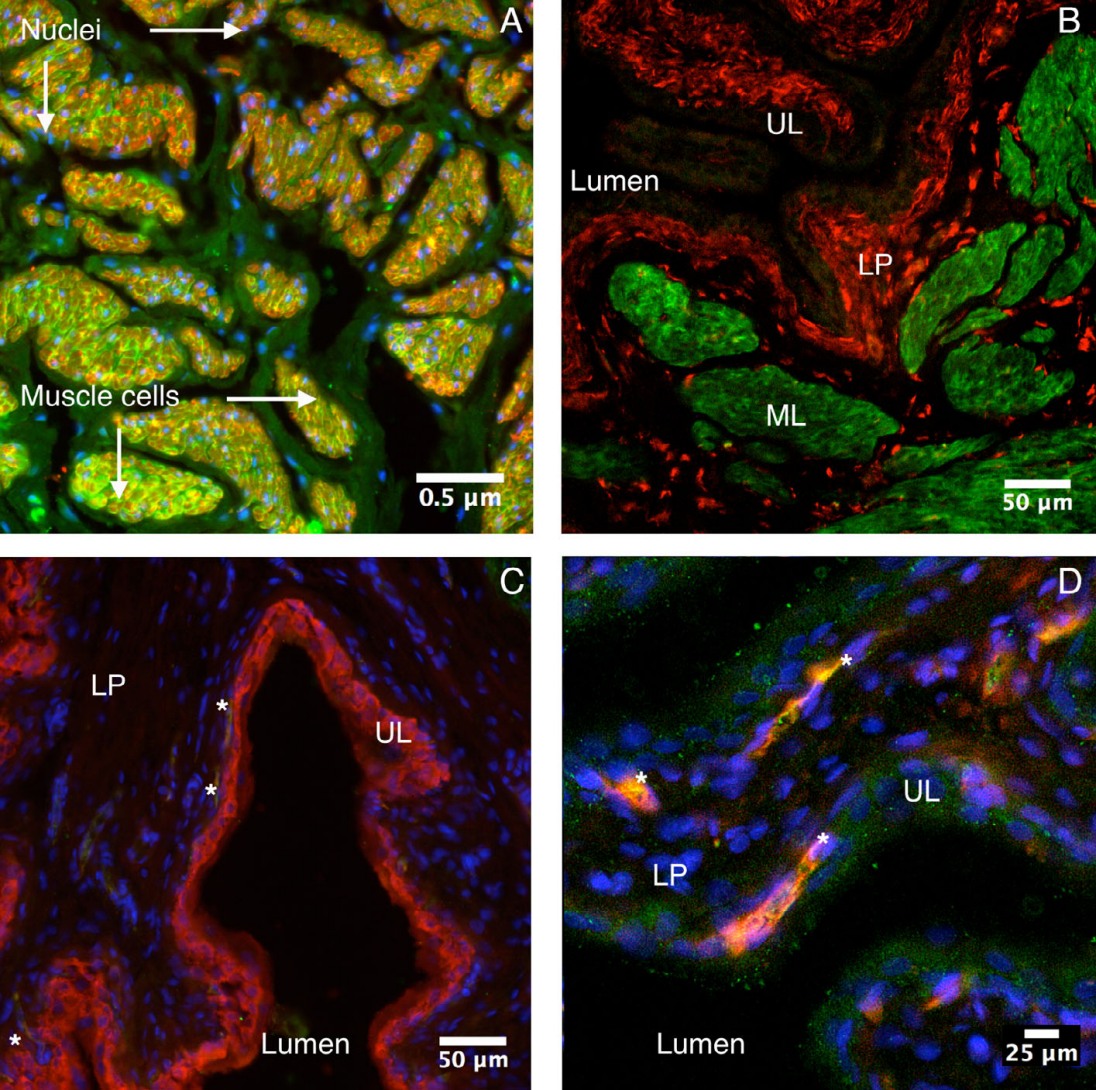
Immunohistochemical double staining of the rat bladder. **(A)** Dystrophin (green) with smooth muscle marker aSMA (red), ×200, Hoechst (blue). Dystrophin was expressed at the sarcolemma, and aSMA was expressed in the cytoplasm of smooth muscle cells. **(B)** Dystrophin (green) with interstitial cell marker VIM (red), ×200. **(C)** Dystrophin (green) with urothelial cell marker PGP 9.5 (red), ×400, Hoechst (blue). **(D)** Dystrophin (green) with neuronal cell marker CGRP (red), ×400, Hoechst (blue). Dystrophin is always shown in green; in all images, there is a weak green signal present in the structure, which, histologically, looks like the urothelium. In close inspection of (**C**), structures (asterisks) can be seen in the suburothelium, which stain positive for dystrophin but do not stain for PGP 9.5. These structures are most likely CGRP^+^ neurons, of which some colocalized (asterisk, **D**) with dystrophin in the LP. aSMA, α‐smooth muscle actin; CGRP, calcitonin gene‐related peptide; LP, lamina propria; ML, smooth muscular layer; PGP 9.5, protein gene product 9.5; UL, urothelial layer; VIM, vimentin. [Color figure can be viewed at wileyonlinelibrary.com]

Colocalization immunochemistry is shown in Figure 2. For each staining, we used an antibody for dystrophin and an additional marker, which stained smooth muscle cells (Fig. 2A), interstitial cells (Fig. 2B), the urothelium (Fig. 2C), or neurons (Fig. 2D). Dystrophin and aSMA were coexpressed in the same cells; that is, the sarcolemma of smooth muscle cells was dystrophin‐positive and the cytoplasm of smooth muscle cells was aSMA‐positive (Fig. 2A). No colocalization was found between dystrophin and interstitial cell maker VIM (Fig. 2B). No clear colocalization was found between dystrophin and urothelial marker PGP 9.5 (Fig. 2C). In all images, there is a weak signal present for dystrophin in the structure that, histologically, looks like the urothelium but does not show clear colocalization with PGP 9.5 (Fig. 2C). Additional AQP staining resulted in a similar staining pattern of the urothelium for AQP‐3 (Fig. 1F). Finally, CGRP immunoreactivity showed neurons in the LP, of which some colocalized with dystrophin (Fig. 2D).

### Developmental Expression of Dystrophin in the Urinary Bladder

**Figure 3:**
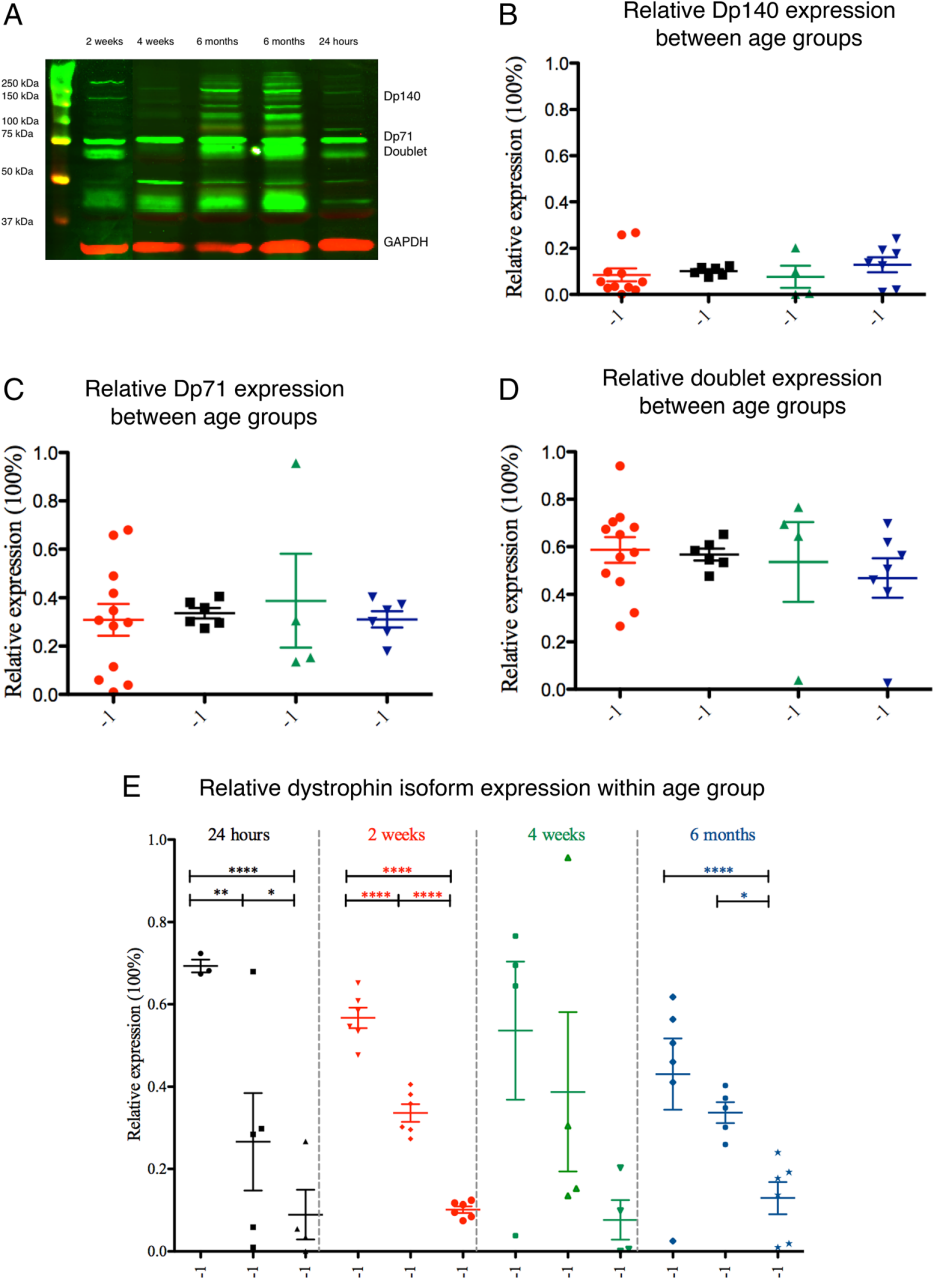
Dystrophin isoform expression levels in the rat bladder during maturation. **(A)** Dystrophin‐immunoreactive bands (green) at ~140 kDa (Dp140), at ~5 kDa (designated Dp71), at ~70 kDa (designated doublet). In this study, we focused on isoforms that were present at all ages, could reliably be quantitated, and are relevant in nonskeletal muscle tissue. Therefore, some additional bands are not analyzed. Immunoreactive bands were quantitated and expressed relative to their respective anti‐GAPDH level (red; ~37 kDa). The membrane was cut between the 2‐week and 4‐week lane because not all age groups were run on the same blot. **(B)** Relative Dp140 expression levels between age groups. **(C)** Relative Dp71 expression levels between age groups. **(D)** Relative doublet expression levels between age groups. **(E)** Relative expression of all 3 isoforms compared within age groups. The doublet showed the highest expression level, followed by Dp71 and by Dp140 (*P < 0.05; **P < 0.01; ****P < 0.0001). All expression levels (mean ± SEM) were calculated relative to total dystrophin expression. Bladders were obtained at postnatal age 24 h (*n* = 11), 2 weeks (*n* = 6), 4 weeks (*n* = 4), or 6 months (*n* = 6). GAPDH, glyceraldehyde‐3‐phosphate dehydrogenase. [Color figure can be viewed at wileyonlinelibrary.com]

Western blots revealed that each sample contained immunoreactive bands at approximately 140 kDa (hereafter called *Dp140*), 75 kDa (hereafter called *Dp71*), as previously reported,^13^ and 2 bands around the molecular weight of 70 kDa (hereafter called *doublet*), which were combined for analysis purposes (Fig. 3A). Two outliers—a doublet in a 24‐h‐old rat sample and a Dp71 immunoreactive band in a sample from a 6‐month‐old rat—were excluded from analysis because they reached a value of more or less than 2 SD of the mean. Total dystrophin expression levels (i.e., the sum of Dp140, Dp71, and doublet expression) did not differ between age groups, and the expression level of each individual isoform did not differ between age groups (Fig. 3B–D).

In the 24‐h age group, the doublet isoform was expressed higher than Dp71, which was expressed higher than Dp140 (doublet vs. Dp71; Dp71 vs. Dp140; Fig. 3E). Similar differences were observed in the 2‐week (doublet vs. Dp71; Dp71 vs. Dp140; Fig. 3E) and 6‐month (Dp71 vs. Dp140; Fig. 3E) age groups. However, in the 4‐week age group, there were no differences in expression levels between the 3 isoforms.

### Dystrophin in Relation to aSMA Expression

**Figure 4:**
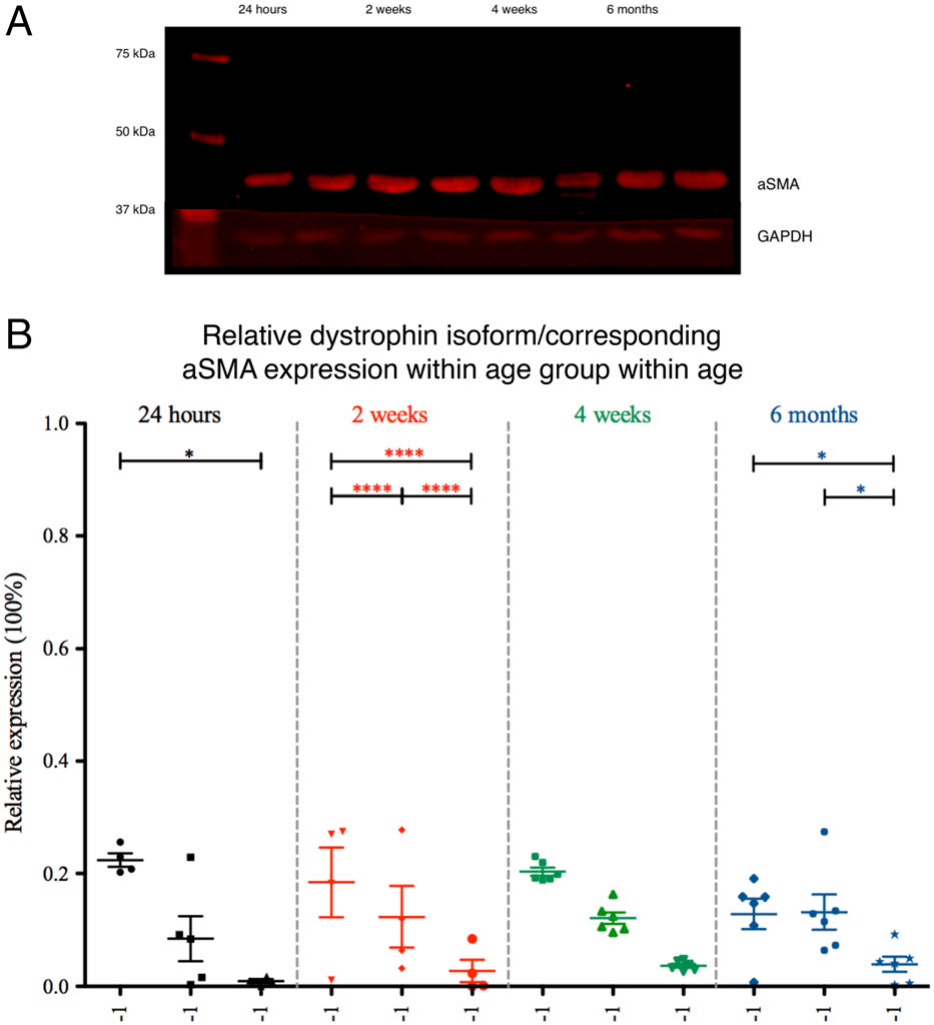
Smooth muscle expression levels in the rat bladder during maturation. **(A)** aSMA‐immunoreactive band at ~42 kDa (red). All immunoreactive bands were quantitated and expressed relative to their respective anti‐GAPDH level (red; ~37 kDa). **(B)** Relative expression of all 3 isoforms corrected for aSMA. Isoform levels corrected for aSMA were in line with the uncorrected results of the dystrophin isoform specific expression (*P < 0.05; **P < 0.01; ****P < 0.0001). All expression levels (mean ± SEM) were calculated relative to total dystrophin expression. Bladders were obtained at postnatal age 24 h (*n* = 5), 2 weeks (*n* = 6), 4 weeks (*n* = 4), or 6 months (*n* = 6). aSMA, α‐smooth muscle actin; GAPDH, glyceraldehyde‐3‐phosphate dehydrogenase. [Color figure can be viewed at wileyonlinelibrary.com]

Western blots revealed an immunoreactive band at the expected protein size of approximately 42 kDa (Fig. 4A). The expression level of aSMA did not differ between age groups, and there was no significant correlation between aSMA expression level and total dystrophin expression (*r* = −0.09, *P* = 0.69). There was also no correlation between a specific isoform and the amount of aSMA (doublet: *r* = 0.03, *P* = 0.87; Dp71: *r* = −.0.25, *P* = 0.25; Dp140: *r* = −.0.33, *P* = 0.14). Finally, the expression level of each isoform was normalized to the amount of aSMA (Fig. 4B). For these aSMA‐normalized dystrophin levels, there was no significant interaction between isoform and age. Within the 24‐h age group, the doublet isoform was expressed higher than Dp140 (doublet vs. Dp140; Fig. 4B). Similar differences were observed in the 2‐week (doublet vs. Dp71; Dp71 vs. Dp140; Fig. 4B) and the 6‐month (Dp71 vs. Dp140 *P* < 0.05; Fig. 4B) age groups. In the 4‐week age group, there were also no statistically significant differences in expression levels between the 3 isoforms. These data are in line with the uncorrected results of the isoform specific expression.

## DISCUSSION

The main findings of this study are that (1) dystrophin is expressed in smooth muscle cells and in afferent nerve fibers, (2) isoform expression levels were unchanged during postnatal development, and (3) there were similar quantitative differences in isoform expression at each age studied, doublet > Dp71 > Dp140. Quantitative changes in muscle mass during development did not influence isoform expression levels.

### Dystrophin Distribution in the Urinary Bladder

The expression of dystrophin in smooth muscle cells of human and rat bladder tissue observed here is in line with previous research showing dystrophin expression in smooth muscle cells of healthy human bladder tissue.^9^ The prostate pathology in the human tissue sample used could result in morphological changes of the bladder (decreased neuronal density, increased epithelial turnover, increased fibrosis in the submucosal layer).^19^ This is why we chose not to quantitate dystrophin expression in these samples but only to verify whether dystrophin was present. Therefore, we postulate that the immunohistochemistry results shown in the rat bladder are translatable to the human situation. In contrast to striated muscle, the role of dystrophin in smooth muscle is less well known. Dystrophin has been described to support smooth muscle actin dynamics that regulate contractile differentiation in mouse aortic smooth muscle cells.^10^ The expression pattern observed in our immunohistochemistry studies suggests that dystrophin is also expressed in blood vessels of the human and rat bladder. The role of the vasculature in bladder function is not entirely clear because it has received relatively little scientific attention. Nevertheless, Tycocki and colleagues^20^ found that myogenic tone in mouse bladder arterioles is attributable to the activity of smooth muscle inward rectifying potassium channels (K_IR_) for the control of bladder blood flow. As dystrophin has been shown to serve as an anchor for K_IR_, specifically K_IR_2.1, in mouse cardiomyocytes,^21^ a similar role for dystrophin may be present in the bladder vasculature.

Furthermore, PGP 9.5 has been demonstrated to stain the urothelium and efferent neurons of mouse urinary bladder,^22^ whereas CGRP identified afferent neurons.^23^ In line with the idea that nerve fibers in the suburothelium are afferent, we observed colocalization of dystrophin with CGRP but not with PGP 9.5. Close inspection of Figure 2C reveals that structures can be seen in the suburothelium, which stain positive for dystrophin but do not stain for PGP 9.5. These structures are most likely afferent CGRP^+^ neurons. This further supports the general finding of many recent studies that LUTS are mostly mediated by afferent system alterations and that direct activation of afferents and the release of urothelially derived mediators play a crucial role in micturition pathophysiology.^24^

The current study showed a weak staining of dystrophin in the urothelial layer but no clear coexpression with PGP 9.5. Dystrophin is possibly involved in water homeostasis in urothelial cells. From a hypothetical point of view, dystrophin could be involved in AQP clustering.^25^, ^26^ In the central nervous system, dystrophin is involved in control of the blood–brain barrier by anchoring glial AQP‐4.^27^ In analogy, dystrophin may have a comparable barrier role in the urothelium. An additional staining of AQP‐3, which has already been shown to be expressed in the urothelium, showed a similar staining pattern which further supports our hypothesis.^25^, ^28^ Because the available AQP‐3 antibody had the same host as the anti‐dystrophin antibody used, causing cross‐reactivity, we were technically not able to support this with colocalization immunohistochemistry.

### Dystrophin Isoform Levels in the Urinary Bladder

We hypothesized that postnatal development goes along with changes in dystrophin isoform expression. Nonetheless, there were no changes in quantitative dystrophin expression between the age groups studied. In all age groups, however, we observed a significantly higher expression level of Dp71 and splice variants than of Dp140.

In the present study, we focused on the quantitation of isoforms that were present at all ages, that could reliably be quantitated, and that have previously been described to be of relevance in nonskeletal muscle tissue. Moreover, the focus in nonskeletal tissue has been on isoforms with a shorter protein size, such as Dp71 and Dp140.^8^ Although the antibody used in our study is also suitable for detection of the full‐length Dp427 in a very similar protocol, we could not consistently show an immunoreactive band at this protein size with the currently used technique.^13^ It is well known that skeletal muscle cells express Dp427 under the control of a tissue and cell specific promotor. We showed dystrophin in the sarcolemma of smooth muscle cells in the bladder with immunohistochemistry; however, with the current techniques, we cannot show the specific isoform.

Furthermore, it is known that several variants of Dp140 and Dp71 exist, which are generated by alternative splicing and produce protein products which differ in protein size.^29^, ^30^ In our study, we also observed additional protein bands, especially in the bladder samples of 6‐month‐old rats in the 71–140 kDa range. Next to multiple splice variants of Dp140, an isoform with a size of 116 kDa is known to exist.^31^ Moreover, a protein band appeared about the size of 40 kDa. Even though an isoform is known with a protein size of 40 kDa, we would not be able to detect this because the C‐terminal that the antibody used is missing in this variant.^32^ Alternative splice variants have been found to differ per tissue type and developmental stage.^33^, ^34^ The appearance of additional protein bands without changes in the quantity of other isoforms suggests additional transcription and translation rather than a shift of isoforms during development.

### Interaction of Dystrophin With Other Proteins

Dystrophin forms the so‐called DGC with other transmembrane proteins to stabilize the plasma membrane in striated muscles. These transmembrane components, dystroglycan and sarcoglycans, in turn connect to the extracellular matrix by means of laminin. Other proteins such as dystrobrevin and syntrophin are connected to the C‐terminal of dystrophin and participate in signal transduction pathways.^7^ The expression of these proteins has been extensively studied in the bladder.^35^, ^36^ In DMD, changes in dystrophin expression are the primary cause of disruption of the DGC.

### Possible Role of Dystrophin in Urological Manifestations of DMD

The present finding of dystrophin expression in afferent nerve fibers of the healthy rat bladder is interesting in relation to the current knowledge of involvement of the afferent system in human micturition physiology and pathological conditions. Afferent neurons play a key role in mediating storage and normal micturition and are also responsible for signaling of bladder filling states.^37^ During the past decade, a shift has been seen from focusing research on contractile mechanisms to focusing on the afferent system, which is an attractive target for pharmacological treatment of various LUTS (i.e., urgency, frequency, nocturia, and urge incontinence).^24^ In analogy to the process taking place in skeletal muscle, a breakdown of the dystrophin protein may cause disturbances in afferent nerve signaling resulting in urological complaints in DMD. The neurological basis of LUTS in DMD remains ambiguous except in cases in which symptoms clearly result from spinal surgery or severe scoliosis due to spinal instability caused by progressive muscle weakness. Peripheral disturbances that have been implicated include skeletal muscle weakness of the pelvic floor, smooth muscle weakness in the gastrointestinal and genitourinary tracts, and functional obstruction during micturition (due to immobilization or constipation) or dysfunctional voiding.^38^ In addition, there may be central disturbances, such as impaired neural control (e.g., dysfunctional clustering of inhibitory γ‐aminobutyric acid type A receptors in the postsynaptic membrane^39^) affecting somatic 38 or autonomic pathways.^40^ Also, it has been hypothesized that upper motor neurons damaged by progressive scoliosis over the course of the disease can result in micturition problems.^41^

### Future Perspectives

Our findings provide sufficient evidence to warrant further research on the role of dystrophin in both smooth muscle functioning and afferent signaling taking isoform variation and function into account. Existing murine models targeting dystrophin involve the classical *mdx* models. Most animals show a mild, nonprogressive phenotype with a mildly reduced lifespan. On the other hand, double‐knockout mice (i.e., utrophin/dystrophin and integrin/dystrophin) present with a phenotype more similar to DMD.^42^ However, a single knockout of integrin shows alterations in bladder function.^43^, ^44^ Therefore, these models seem less suitable to study the role of dystrophin in micturition. An alternative is a smooth muscle‐specific knockout of dystrophin. Thus, the functional role of dystrophin deficiency could be investigated in a murine model by means of behavioral as well as cystometric measurements.^45^

Urological evaluation and urodynamic investigations of LUTS in patients may further increase insights into the pathophysiology. Our findings highlight the importance of urology in multidisciplinary management of DMD and may increase LUTS recognition. Moreover, the results of this study emphasize that micturition problems in DMD may have not only a myogenic but also an afferent neurogenic origin.

In conclusion, dystrophin is expressed by smooth muscle cells and afferent nerve fibers in the healthy rat bladder. Dp71 and splice variant expressions are two‐ to threefold higher than those of Dp140, independently of age. The presence of dystrophin in the bladder makes this observation interesting and useful in future research. This may eventually open a new therapeutic window to treat LUTS in pathological conditions such as DMD. However, the exact pathophysiology of LUTS in DMD remains to be determined.

A selection of this data was presented at the XXIII World Congress of Neurology, September 2017, Kyoto, Japan; and at the World Muscle Society Congress, October 2018, Mendoza, Argentina.

The authors thank Tom A. T. Marcelissen, MD, PhD for the human bladder tissue samples; Blanche Schroen, MD, PhD, Robin H. K. O. Verjans, MSc, and Britt T. J. van Hagen, PhD for the rat tissue used in this study; Jan Beckervordersandforth, MD, PhD for the aSMA antibody; Hellen E. Steinbusch for the microscopy and staining assistance; and V. Limapassos for the statistical advice.

Ethical Publication Statement: We confirm that we have read the Journal's position on issues involved in ethical publication and affirm that this report is consistent with those guidelines.

## Supporting information


**Supplementary data S1** Overview of different dystrophin isoforms along with the epitope targeted by the antibody (Ab15277, Abcam, Cambridge, UK).
