# Supplementary figures and images for: Dystrophin is expressed in smooth muscle and afferent nerve fibers in the rat urinary bladder
Source: Muscle Nerve. 2019 Jun 7;60(2):202–10. doi: 10.1002/mus.26518 (PMC6771971; doi:10.1002/mus.26518)

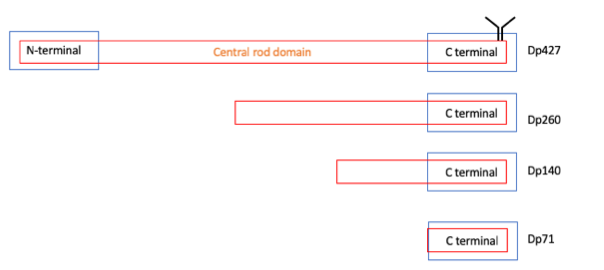

Supplement: Supplementary file 1 — Supplementary data S1 Overview of different dystrophin isoforms along with the epitope targeted by the antibody (Ab15277, Abcam, Cambridge, UK). [file MUS-60-202-s001.docx]
